# Acoustic Identification of the Voicing Boundary during Intervocalic Offsets and Onsets based on Vocal Fold Vibratory Measures

**DOI:** 10.3390/app11093816

**Published:** 2021-04-23

**Authors:** Jennifer M. Vojtech, Dante D. Cilento, Austin T. Luong, Jacob P. Noordzij, Manuel Diaz-Cadiz, Matti D. Groll, Daniel P. Buckley, Victoria S. McKenna, J. Pieter Noordzij, Cara E. Stepp

**Affiliations:** 1 Department of Biomedical Engineering, Boston University, Boston, MA 02215, USA; 2 Department of Speech, Language, and Hearing Sciences, Boston University, Boston, MA 02215, USA; 3 Delsys, Inc. and Altec, Inc., Natick, MA, 01760, USA; 4 Department of Otolaryngology – Head and Neck Surgery, Boston University School of Medicine, Boston, Massachusetts, USA

**Keywords:** relative fundamental frequency, high-speed videoendoscopy, voice assessment, laryngeal tension

## Abstract

Methods for automating relative fundamental frequency (RFF)—an acoustic estimate of laryngeal tension—rely on manual identification of voiced/unvoiced boundaries from acoustic signals. This study determined the effect of incorporating features derived from vocal fold vibratory transitions for acoustic boundary detection. Simultaneous microphone and flexible nasendoscope recordings were collected from adults with typical voices (N=69) and with voices characterized by excessive laryngeal tension (N=53) producing voiced–unvoiced–voiced utterances. Acoustic features that coincided with vocal fold vibratory transitions were identified and incorporated into an automated RFF algorithm (“aRFF-APH”). Voiced/unvoiced boundary detection accuracy was compared between the aRFF-APH algorithm, a recently published version of the automated RFF algorithm (“aRFF-AP”), and gold-standard, manual RFF estimation. Chi-square tests were performed to characterize differences in boundary cycle identification accuracy among the three RFF estimation methods. Voiced/unvoiced boundary detection accuracy significantly differed by RFF estimation method for voicing offsets and onsets. Of 7721 productions, 76.0% of boundaries were accurately identified via the aRFF-APH algorithm, compared to 70.3% with the aRFF-AP algorithm and 20.4% with manual estimation. Incorporating acoustic features that corresponded with voiced/unvoiced boundaries led to improvements in boundary detection accuracy that surpassed the gold-standard method for calculating RFF.

## 1. Introduction

Excessive and/or imbalanced laryngeal muscle forces have been implicated in over 65% of individuals with voice disorders [1]. The specific pathophysiology of laryngeal hypertonicity is a known characteristic of many functional, structural, and neurological disorders, including: adductor-type laryngeal dystonia [2, 3], hyperfunctional voice disorders [e.g., muscle tension dysphonia, nodules; 4], and Parkinson’s disease [5]. Despite this prevalence, current clinical voice assessments fall short in objectively quantifying the degree of laryngeal muscle tension. For instance, auditory-perceptual judgments are a gold-standard technique used to assess voice quality, but the reliability and validity of these judgments remains questionable [6, 7]. Likewise, manual laryngeal palpation techniques can be useful for evaluating tension of the extrinsic laryngeal and other superficial neck musculature; however, these methods do not assess the intrinsic laryngeal muscles and, moreover, are subject to the skill and experience of the practitioner [8]. Much of the research surrounding laryngeal hypertonicity has therefore turned to acoustic analyses, for which data can be non-invasively collected via a microphone. Acoustic signals can provide insight into characteristics of the glottal source (e.g., timing, frequency, and amplitude of vocal fold vibration). To date, however, a single acoustic indicator specific to laryngeal muscle tension has not been identified.

**Figure 1.**
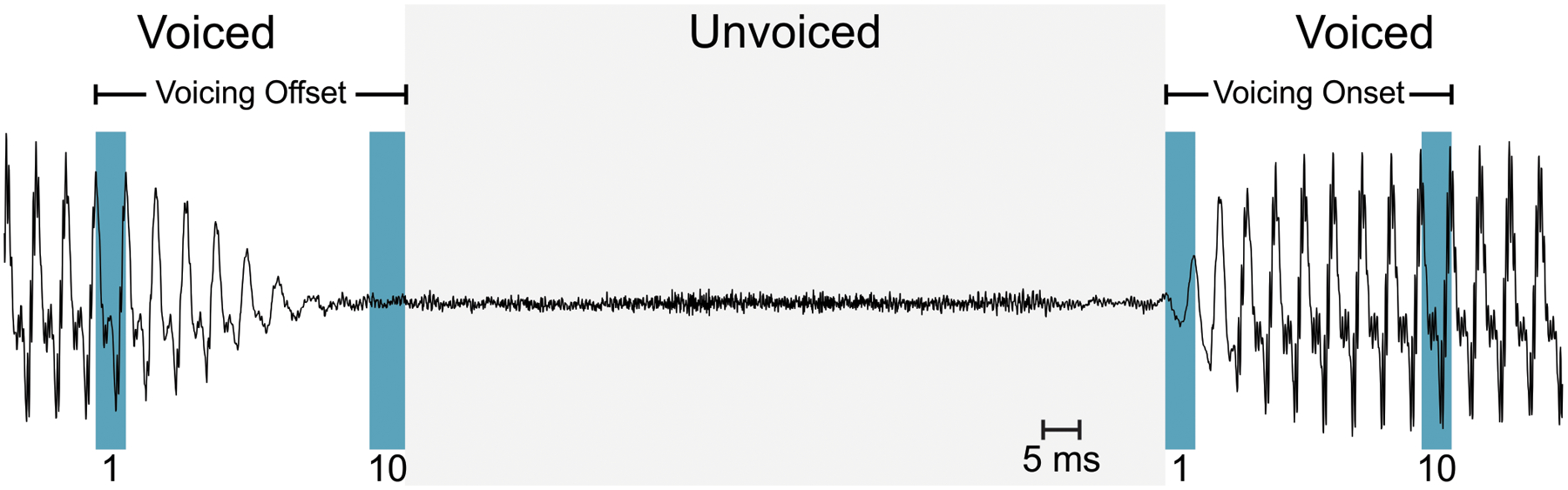
Acoustic waveform of the vowel–voiceless consonant–vowel production, /ifi/. Voicing cycles preceding the voiceless consonant, /f/, are marked as voicing offset cycles, whereas those following the /f/ are indicated as voicing onset cycles. The first and tenth vocal cycles are highlighted for each transition.

In recent years, relative fundamental frequency (RFF) has been suggested as an acoustic indicator of laryngeal muscle tension. Estimated from short-term changes in instantaneous *f*_*o*_ during intervocalic offsets and onsets, RFF is a non-invasive, objective measure that shows promise in estimating the degree of baseline laryngeal muscle tension. RFF can be calculated from a vowel–voiceless consonant–vowel (VCV) production as in Fig. 1. The instantaneous *f*_*o*_ of the ten voiced cycles preceding (“voicing offset”) and following (“voicing onset”) the voiceless consonant are each estimated and normalized to a steady-state *f*_*o*_ of the nearest vowel $\left(f_{o}^{\;ref}\right)$ to produce an RFF estimate in semitones (ST):

(1) $$\text{RFF }\left(\text{ST}\right){\text{ = 12×log}}_{\text{2}}\left(\frac{f_{o}}{f_{o}^{\;ref}}\right)$$

Recent work exploring the clinical utility of RFF for assessing laryngeal muscle tension suggests that RFF correlates with severity of vocal symptoms in speakers with dysphonia [9–11] and can distinguish speakers with and without voice disorders characterized by excessive laryngeal muscle tension, including individuals with vocal hyperfunction (VH) [9, 12, 13], Parkinson’s disease [14, 15], and adductor-type laryngeal dystonia [11]. Specifically, those with voice disorders characterized by excessive laryngeal muscle tension tend to exhibit lower average RFF values, perhaps due to increased baseline laryngeal muscle tension that impedes their ability to leverage tension as a strategy for devoicing (voicing offset) and reinitiating voicing (voicing onset). RFF also normalizes (increases) in individuals with VH following voice therapy [i.e., a functional change; 9, 16], but not in individuals with vocal nodules or polyps following therapy [i.e., structural intervention; 16]. This suggests that RFF reflects functional voice changes rather than structural voice changes. It has also been demonstrated that RFF captures within-speaker changes in vocal effort [17], or the perceived exertion of a vocalist to a perceived communication scenario [i.e., vocal demand; 18], as well as indirect kinematic estimates of laryngeal tension [19]. Despite the promise of RFF as an acoustic estimate of laryngeal muscle tension, this measure requires refinement before it will be appropriate for routine clinical use.

RFF can currently be calculated in two ways: manually or semi-automatically. The current gold-standard method of computing RFF is through manual estimation techniques using Praat software [20]. Due to the time- and training-intensive procedures that are necessary to reliably perform manual RFF estimation, a semi-automated RFF algorithm, called the *aRFF* algorithm was developed [21, 22]. Both manual and aRFF estimation techniques use autocorrelation to estimate *f*_*o*_, which occurs by comparing a segment of the voice signal with itself when offset by a certain period. Despite being a relatively fast *f*_*o*_ estimation method, autocorrelation assumes signal periodicity and *f*_*o*_ stationarity, requiring 2–3 complete pitch periods to examine the physiological *f*_*o*_ ranges encountered in speech [23]. These characteristics are not ideal for estimating *f*_*o*_ during the voicing offset and onset transitions examined in RFF, which specifically capture rapid changes in *f*_*o*_. Indeed, Vojtech, et al. [24] compared the effects of different *f*_*o*_ estimation techniques on resulting RFF estimates, determining that *f*_*o*_ estimation via the Auditory-SWIPE′ algorithm (an algorithm that estimates the *f*_*o*_ of an input sound by first filtering the sound using a auditory-processing front-end then comparing the spectrum of the filtered sound to a sawtooth waveform) [25] led to smaller errors between manual and semi-automated RFF estimates compared to autocorrelation. The results of this work led to a refined version of the aRFF algorithm that employs Auditory-SWIPE′ for *f*_*o*_ estimation, as well as using the acoustic metric, pitch strength [26], to account for differences in voice sample characteristics (e.g., recording location, overall severity of dysphonia). Incorporating both **A**uditory-SWIPE′ and **p**itch strength-based sample categories, this algorithm is called *aRFF-****AP***.

In both manual and semi-automated RFF estimation methods, the most tedious step of the RFF computational process is identifying the boundary between voiced and unvoiced speech. As RFF depends on the termination and initiation of voicing within a VCV production, these points in time must be identified from the acoustic signal prior to collecting vocal cycles for RFF estimation. Manual RFF estimation relies on trial-and-error techniques of trained technicians to locate this boundary [requiring 20–40 minutes of analysis time per RFF estimate; 11], whereas the semi-automated RFF algorithms (aRFF, aRFF-AP) take advantage of a faster, more objective approach. Specifically, three acoustic features (normalized peak-to-peak amplitude, number of zero crossings, and waveform shape similarity) are examined in time to identify where a state transition in feature values occurs to locate voiced/unvoiced boundary. The aRFF and aRFF-AP algorithms assume that each acoustic feature will exhibit a substantial change in feature values over time and that this change will occur at the boundary between voiced and unvoiced segments.

The methodology used to identify the voiced/unvoiced boundary in semi-automated RFF estimation requires further inquiry. First, it is unclear as to whether the three features used in aRFF and aRFF-AP algorithms are the best choice of acoustic features to mark the initiation and termination of vibration since voiced/unvoiced boundary detection accuracy has not been formally assessed amongst the three features or compared to that of other acoustic features (e.g., cepstral peak prominence). Second, there is uncertainty in how the boundary identified using these acoustic features corresponds to the physiological initiation or termination of vocal fold vibration. This is because both manual and semi-automated RFF methods rely only on the acoustic signal, which only provides indirect information about the vibration of the vocal folds and may be masked by supraglottic resonances, coarticulation (e.g., due to concurrent aspiration and frication), and radiation [27]. Thus—in addition to a lack of *f*_*o*_ stationarity during vocal fold offset and onset transitions—signal masking adds to the complexity of identifying the initiation or termination of vocal fold vibration. The uncertainties in acoustic boundary cycle identification warrant further investigation to (i) inform the implementation of acoustic features used in the semi-automated RFF algorithm and (ii) validate manual RFF estimation as a gold-standard that accurately represents changes in instantaneous *f*_*o*_ during voicing offsets and onsets.

High-speed videoendoscopy (HSV) may be a useful technique to examine the relationship between the acoustic signal and vocal fold vibration. By sampling at frame rates much greater than typical modal (i.e., the vocal register most typically used during conversational speech) *f*_*o*_ values, HSV can capture cycle-to-cycle changes in vocal fold vibratory behavior during voicing offsets and onsets [28–30]. Indeed, prior work has employed HSV to investigate voicing offsets and onsets relative to the acoustic signal: Patel, et al. [31] acquired simultaneous recordings via a microphone and rigid laryngoscope as vocally healthy speakers repeated /hi hi hi/ at their typical pitch and loudness. The results of this work indicated a tight coupling between the acoustic signal and the physiological vibrations of the vocal folds; however, this relationship may not be generalizable to the acoustic outputs typically examined with RFF. Transitioning between a vowel and the voiceless glottal fricative, /h/, may require different mechanisms than when transitioning between a vowel and a voiceless obstruent produced via oral constrictions (e.g., /f/, /s/, /ʃ/, /p/, /t/, /k/). For instance, Löfqvist, et al. [32] observed that glottal vibrations continued uninterruptedly through the /h/ during the production of /aha/ sequences by some speakers; these vibrations were not present for voiceless consonant productions of /asa/ or /apa/. Such differences could ultimately affect the relationship between oscillatory events obtained from the laryngoscopic images and from the acoustic signal. Additionally, the participants in Patel, et al. [31] were limited to adults with typical voices, whereas the target population for employing RFF in clinical voice assessments includes speakers with voice disorders characterized by excessive laryngeal muscle tension. As such, additional investigations are needed to examine voicing offsets and onsets in the context of speakers with and without voice disorders.

To carry out the present investigation, speakers with typical voices and speakers with voices characterized by excessive laryngeal muscle tension were enrolled across a wide age range to investigate the relationship between acoustic features and vocal fold vibratory characteristics during intervocalic voicing offset and onsets. Acoustic features were identified that corresponded with the physiological initiation and/or termination of vocal fold vibration. The aRFF-AP algorithm was then further refined by modifying algorithmic parameters corresponding to the **H**SV-tuned acoustic feature set (“aRFF-AP**H**”). Voiced/unvoiced boundary detection accuracy was computed for each of the three RFF methods (manual estimation, aRFF-AP, aRFF-APH) relative to the actual vocal fold vibratory features identified via HSV. It was hypothesized that incorporating features related to the onset and offset of vocal fold vibration would improve the accuracy of acoustic voiced/unvoiced boundary detection (aRFF-APH) over methods that did not leverage these tuned features (manual, aRFF-AP).

## 2. Materials and Methods

### 2.1 Participants

Sixty-nine individuals with typical voices (33 cisgender females, 36 cisgender males) aged 18–91 years (*M* = 43.2 years, *SD* = 23.1 years) were enrolled in the study. All provided informed, written consent in compliance with the Boston University Institutional Review Board. All were fluent in English and had no history of speech, language, hearing, neurological, or voice problems. A certified, voice-specializing speech-language pathologist screened all participants with typical voices for healthy vocal function via auditory-perceptual assessment and flexible nasendoscopic laryngeal imaging.

Fifty-three individuals with voice disorders characterized by excessive laryngeal tension (28 cisgender females, 1 transgender female, 23 cisgender males, 1 transgender male; *M* = 49.5 years, *SD* = 18.4 years, *range* = 19–75 years) were enrolled in the study. All provided informed, written consent in compliance with the Boston University Institutional Review Board. All were fluent in English and reported no history of hearing problems. Participants within this group were either diagnosed with idiopathic Parkinson’s disease (PD) by a neurologist, or were diagnosed with a hyperfunctional voice disorder (HVD; e.g., muscle tension dysphonia) by a board-certified laryngologist. All individuals with PD were recorded while on their typical carbidopa/levodopa medication schedule. Individuals who used deep brain stimulation devices (N = 5) were requested to turn their device off for the duration of the data collection. Of the 53 participants with voice disorders, 25 (6 cisgender females, 1 transgender female, 18 cisgender males) were diagnosed with PD. The average time since diagnosis was 7 years (*SD* = 5.8 years, *range* = 0–24 years), and the average severity of motor complications as assessed via the Movement Disorder Society-sponsored revision of the Unified Parkinson’s Disease Rating Scale (Part III) were moderate (*M* = 48.8, *SD* = 20.5, *range* = 13–91). The remaining 28 participants (22 cisgender females, 5 cisgender males, 1 transgender male) were diagnosed with HVDs, including muscle tension dysphonia (20/28), vocal fold nodules (4/28), vocal fold polyp (2/28), vocal fold scarring (1/28), and hyperdermal lesion with secondary supraglottic compression (1/28).

A speech-language pathologist specializing in voice disorders assessed the overall severity of dysphonia (OS; 0–100) of each participant using the Consensus Auditory-Perceptual Evaluation of Voice (CAPE-V). The average OS for participants with typical voices was 8.3 (*SD* = 6.7, *range* = 0.6–34.2), and that of participants with either an HVD or PD was 15.6 (*SD* = 12.4, *range* = 0.9–51.3). The speech-language pathologist rerated 15% of participants in a separate sitting to ensure adequate intrarater reliability. The Pearson’s product-moment correlation coefficient was calculated on the ratings using the statistical package R (Version 3.2.4), yielding an intrarater reliability of *r* = .96. The overall demographic information for participants with typical voices (split into young adults < 40 years of age and older adults ≥ 40 years of age), participants with HVDs, and participants with PD are included in Table 1.

### 2.2 Procedure

The current study comprised three main components: participant training, experimental setup, and data collection. In the training segment, each participant was instructed to produce the speech tokens that would be simultaneously captured via microphone and flexible nasendoscope during data collection. Following the training segment, participants were instrumented with recording equipment. Data collection then commenced, during which participants were cued to produce speech tokens during a nasendoscopic examination that totaled approximately 5–10 minutes. The overall experimental time (including consent, training, setup, and recording) required approximately 1–2 hours.

#### 2.2.1 Participant Training

Participants were trained to produce eight iterations of the VCV utterance, /ifi/. This token was selected since the phoneme /i/ provides an open pharynx to better view the vocal folds under endoscopy [19], whereas the phoneme /f/ has been shown to minimize within-speaker variations in RFF [33]. Each participant was instructed to produce four /ifi/ utterances, take a breath, and then produce the remaining four /ifi/ utterances.

Participants were then trained to produce the eight /ifi/ utterances at different vocal rates and levels of vocal effort. These modifications were chosen to alter the stiffness of the laryngeal musculature [34] to, in turn, produce voice with varying degrees of laryngeal muscle tension. Using methodology employed by McKenna, et al. [19], a metronome was used to train three vocal speeds: slow rate (50 beats per minute), regular rate (65 beats per minute), and fast rate (80 beats per minute). Similarly, participants were cued using methodology described by McKenna, et al. [19] to produce voice with varying levels of vocal effort (mild effort, moderate effort, maximum effort) while maintaining comfortable speaking rate and volume. While instructing participants to “increase your effort during your speech as if you are trying to push your air out,” mild effort was cued as “mildly more effort than your regular speaking voice,” moderate effort as “more effort than mild,” and maximum effort as “as much effort as you can while still having a voice.”

#### 2.2.2 Experimental Setup

After the training segment, participants were seated in a sound-attenuated booth and instrumented with recording equipment. This included a directional headset microphone (Shure SM35 XLR) placed 45° from the midline and 7 cm from the lips, as well as a neck-surface accelerometer (BU series 21771 from Knowles Electronic, Itasca, IL) that was placed on the anterior neck, superior to the thyroid notch and inferior to the cricoid cartilage using double-sided adhesive. Microphone and accelerometer signals were pre-amplified (Xenyx Behringer 802 Preamplifier) and digitized at 30 kHz (National Instruments 6312 USB).

A flexible routine endoscope (Pentax, Model FNL-10RP3, 3.5-mm) was then passed transnasally through the inferior nasal turbinate, superior to the soft palate, and into the hypopharynx for laryngeal visualization. In cases in which participant nasal anatomy or reported discomfort interfered with image acquisition using the routine endoscope, a flexible slim endoscope (Pentax, Model FNL-7RP3, 2.4-mm) was used. A numbing agent was not administered so as to not affect laryngeal function [35], but a nasal decongestant was offered prior to insertion to minimize participant discomfort as the endoscope was passed through the nasal cavity. To record images of the larynx, the endoscope was attached to a camera (FASTCAM Mini AX100l; Model 540K-C-16GB; 256 × 256 pixels) with a 40-mm optical lens adapter. A steady xenon light was used for imaging (300 W KayPENTAX Model 7162B).

#### 2.2.3 Experimental Recording

During the endoscopy procedure, participants were instructed to produce the eight ifi/ repetitions for each condition, which were cued in the following order: slow rate, regular rate, fast rate, mild effort, moderate effort, and maximum effort. Participants completed a minimum of two recordings per condition, and recordings were repeated when the experimenter determined that the vocal folds were not adequately captured (e.g., obstruction by the epiglottis). Video images were acquired at a frame rate of 1 kHz using Photron Fastcam Viewer software (v.3.6.6) to track the fundamental frequency of vibration of the vocal folds, which is estimated to be 85–255 Hz during modal phonation in adults [36]. Recording was triggered by the camera software and a custom MATLAB (version 9.3; The MathWorks, Natick, MA) script that automatically time-aligned the video images with the microphone and accelerometer signals. Due to the recording limitations of the high-speed imaging system, the synchronized microphone, accelerometer, and HSV recordings were restricted in duration to 7.940 seconds when the 3.5-mm endoscope was used and 8.734 seconds when the 2.4-mm endoscope was used.

### 2.3 Data Analysis

#### 2.3.1 High-speed Video Processing

##### 2.3.1.1 Technician Training

A semi-automated algorithm was used to identify the physiological termination and initiation of vocal fold vibration from each /ifi/ production. To carry out this processing, a series of technicians used the algorithm to compute the glottic angle waveform, from which vocal fold abductory and adductory patterns were isolated. The training and experimental data processing schemes used to extract vocal fold vibratory features are described in detail below.

Prior to processing experimental data, nine technicians underwent a training scheme described by McKenna, et al. [37]. In brief, technicians were first trained to measure glottic angles (extending from the anterior commissure along the medial vocal fold edge to the vocal process) from images obtained during a flexible nasendoscopic procedure using a halogen light source and acquired at a conventional framerate of 30 frames per second. Technicians were required to meet two-way mixed-effects intraclass correlation coefficients (ICC) for consistency of agreement ≥ .80 when compared to glottic angle markings made previously by a gold-standard technician [38]. The average reliability for the nine technicians was *ICC(3,1)* = .89 (*SD* = .01, *range* = .88–.91).

The nine technicians then completed training to use a semi-automated glottic angle tracking algorithm, as described in detail in Diaz-Cadiz, et al. [38]. Using this algorithm, the technicians were trained to use time-aligned microphone, accelerometer, and video frames captured during an /ifi/ utterance to semi-automatically estimate the glottic angle over time. Within the glottic angle tracking training, technicians were required to meet agreement standards of *ICC(3,1)* ≥ .80 compared to a gold-standard technician, described in Diaz-Cadiz, et al. [38]. The resulting average reliability of the nine technicians was *ICC(3,1)* = .85 (*SD* = .04, *range* = .80–.91).

##### 2.3.1.2 Experimental Data Processing

To process experimental data, technicians first determined whether each /ifi/ production was analyzable based on manual inspection of the laryngoscopic recordings. An /ifi/ production was rejected from further analysis if the glottis was obstructed (e.g., by the epiglottis), if video quality was too poor to resolve the glottis, or if an /ifi/ production at the end of the recording was incompletely captured due to the pre-defined recording length. Of the potential 9172 /ifi/ productions recorded, 12.8% were considered unusable (1173 of 9172), leaving 7999 for further processing.

Technicians then used the semi-automated angle algorithm to calculate the glottic angle waveform for the usable /ifi/ productions (*N* = 7999). Within this analysis, each of the nine technicians was assigned to analyze a subset of the 122 speakers, wherein the assigned technician processed all /ifi/ productions of each speaker within the subset. For each speaker, the assigned technician determined whether the /ifi/ production was usable and, if so, obtained a quantitative estimate of the glottic angle for the production. Manual intervention was implemented if algorithmic estimates of the glottic angle waveform was deemed inappropriate by the technician; if errors persisted following manual intervention, the technicians were instructed to mark the instance as unusable. The technicians accepted the fully automated results in 75.0% of cases (6000 of 7999), whereas the technicians accepted the automated results only after performing manual glottic angle intervention in 21.5% of cases (1721 of 7999). The remaining 3.5% of cases were discarded due to producing inappropriate glottic angle estimates even after manual-assisted angle estimation (278 of 7999). This analysis resulted in 7721 usable /ifi/ productions for further processing. This initial data processing was then rechecked by a second technician.

**Figure 2.**
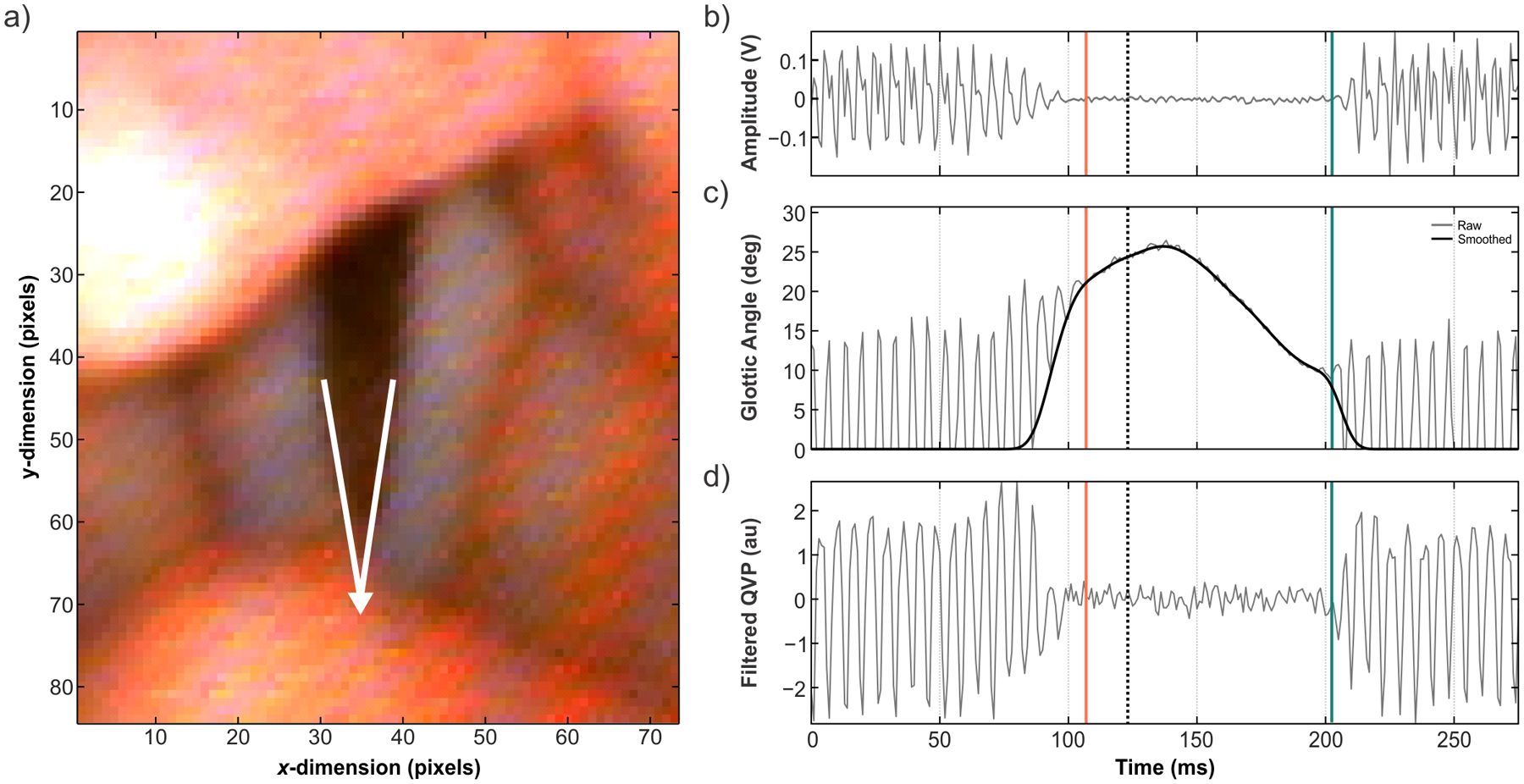
(**a**) View of the vocal folds under flexible nasendoscopy, with the glottic angle marked from the anterior commissure to the vocal processes, (**b**) acoustic signal, (**c**) raw glottic angle waveform (gray) with smoothed data overlay (black), and (**d**) Filtered quick vibratory profile (QVP). The black dotted line in (**b**), (**c**), and (**d**) indicates the current video frame shown in (**a**). The time of voicing offset (orange solid line) and time of voicing onset (teal solid line) are indicated in (**b**), (**c**), and (**d**).

A series of kinematic time points were then extracted from each usable /ifi/ production to mark the physiological termination or initiation of vocal fold vibration. Technicians were presented with a MATLAB GUI showing time-aligned high-speed video frames, the microphone signal, the previously extracted glottic angle waveform, and a high-pass filtered version of the quick vibratory profile (QVP; see Fig. 2). The QVP was included here as an alternative to the glottic angle waveform due to its sensitivity to HSV imagery and superior ability to track the vibrating glottis during the transition between voiced and unvoiced segments [29]. The QVP was calculated by (i) centering the HSV frame over the glottis using the semi-automated glottic angle extraction algorithm from Diaz-Cadiz, et al. [38], (ii) calculating vertical and horizontal profiles of the HSV frames using the methodology from Ikuma, et al. [29], and (iii) high-pass filtering the resulting QVP using a 7^th^ order Butterworth filter to attenuate low frequency energy below a cut-off frequency of 50 Hz.

With the MATLAB GUI, a total of three technicians used the time-aligned microphone signal, glottic angle waveform, and QVP to identify the time of voicing offset (t_off_) and the time of voicing onset (t_on_). For each participant, a single technician was assigned to identify t_off_ and t_on_ for all utterances. In this analysis, t_off_ was described as the termination of the last vibratory cycle before the voiceless consonant, whereas t_on_ was characterized as the initiation of the first vibratory cycle after the voiceless consonant. In the event that the vocal folds exhibited an abrupt closure at the start of voicing onset (i.e., prior to vocal fold vibration), t_on_ was extracted as the time point immediately before the point of abrupt vocal fold closure. Technicians were instructed to use the glottic angle waveform and QVP to identify these two time points, then corroborate the selected indices via manual visualization of the raw HSV images. This process was carried out to minimize errors that may occur if the glottic angle waveform failed to capture small glottal gaps during vibratory cycle phases or if the QVP was confounded by lighting artifacts (e.g., intensity saturation due to the epiglottis coming into view). The microphone signal was included within the GUI to orient technicians in the event that the glottic angle waveform and QVP both failed to properly track the vibrations of the vocal folds; in such cases, the technicians were instructed to indicate that the production needed to be rejected or reprocessed.

The technicians each reanalyzed 10% of participants in a separate sitting to ensure adequate intrarater reliability. The three technicians also analyzed the HSV images of the same participant to assess interrater reliability. Intrarater reliability was assessed via two-way mixed-effects ICCs for absolute agreement, whereas interrater reliability was computed using two-way mixed-effects ICCs for consistency of agreement (single measures). Intrarater reliability ranged from .86 to .99, with an overall mean reliability of .96 (*SD* = .05). Average interrater reliability was *ICC(3,1) =* .91 (*95% CI* = .86–.96) for t_off_ and *ICC(3,1) =* .97 (*95% CI* = .96–.99) for t_on_.

#### 2.3.2 Manual RFF Estimation

Five technicians were trained^1^ to manually estimate RFF using methodology described in Vojtech and Heller Murray [39]. Table 2 shows the number of participants that each of the five technicians rated throughout the course of data collection. Two trained technicians carried out manual RFF estimation on each participant (7721 total /ifi/ productions). Mean RFF values were computed across two technicians to use as the gold-standard for RFF estimates.

Intrarater reliability was assessed via Pearson correlation coefficients within each technician when instructed to reanalyze 20% of participants in a separate sitting, whereas interrater reliability was computed via two-way mixed-effects ICCs for consistency of agreement. The average intrarater reliability was calculated as *r* = .90 (*SD* = .05, *range* = .84–.97), and the average interrater reliability was computed as *ICC(3,1)* = .93 (*SD* = .04, *range* = .87–.98). Rater reliability was also examined by assessing the difference between selected boundary cycles (i.e., voicing offset cycle 10, voicing onset cycle 1) of original and reanalyzed samples. The mean intrarater error was 0.64 vocal cycles (*SD* = 0.44 cycles, *range* = 0–5 cycles), and the mean interrater error was 0.71 vocal cycles (*SD* = 0.41 cycles, *range* = 0–6 cycles).

#### 2.3.3 Semi-automated RFF Estimation

Semi-automated RFF estimation was performed on all 7721 /ifi/ productions using the MATLAB-based aRFF-AP algorithm, which is described in detail in Vojtech, et al. [24]. The aRFF-AP algorithm estimates RFF using a 9-step process that includes: (1) identifying the voiceless consonant and vowels in each production via high-to-low energy ratios of the acoustic signal, (2) manually confirming and/or modifying the locations of the voiceless consonant and vowels in the acoustic signal, (3) estimating the average *f*_*o*_ and pitch strength of the vowels via the Auditory-SWIPE′ algorithm [25], (4) categorizing the voice signal based on average pitch strength, (5) identifying peaks and troughs of potential vocal cycles pertaining to the vowel, (6) estimating a series of acoustic features during the transition into or out of the voiceless consonant, (7) locating the boundary between each vowel and the voiceless consonant by applying category-based thresholds to the acoustic feature vectors, (8) rejecting instances that do not meet specified criteria (e.g., less than 10 onset or offset cycles, glottalization, misarticulation, voicing during the voiceless consonant), and (9) calculating RFF according to Eq. 1.

The relationship between acoustic and physiologic voicing transitions was assessed by examining acoustic features relative to the termination (t_off_) or initiation (t_on_) of voicing. This step is distinct from the development of the aRFF-AP algorithm, as Vojtech, et al. [24] examined acoustic features relative to the intervocalic voicing transitions indicated by manual RFF estimation. To perform this analysis, a literature review was conducted to select a set of acoustic features that showed promise in distinguishing voiced and unvoiced segments, as is the goal of the acoustic features in the semi-automated RFF algorithm (i.e., step 7 of the aRFF-AP algorithm). The acoustic features that best corresponded with the termination or initiation of voicing were then implemented in the aRFF-AP algorithm.

##### 2.3.3.1 Acoustic Feature Selection

In the aRFF-AP algorithm, acoustic feature trends are examined to identify a state transition in feature values that mark the *boundary cycle*, or the vocal cycle that distinguishes the vowel from the voiceless consonant. The boundary cycle is offset cycle 10 for voicing offset and onset cycle 1 for voicing onset (see Fig. 1). The aRFF-AP algorithm uses three acoustic features to characterize the transition between voiced and unvoiced segments: normalized peak-to-peak amplitude, number of zero crossings, and waveform shape similarity.

In addition to the three features included within the aRFF-AP algorithm, a set of 15 new acoustic features were examined with respect to classifying voiced and unvoiced speech segments: (1) autocorrelation, (2) mean cepstral peak prominence, (3) average pitch strength, (4) average voice *f*_*o*_, (5) cross-correlation, (6) low-to-high ratio of spectral energy, (7) median pitch strength, (8) median voice *f*_*o*_, (9) normalized cross-correlation, (10) short-time energy, (11) short-time log energy, (12) short-time magnitude, (13) signal-to-noise ratio, (14) standard deviation of cepstral peak prominence, and (15) standard deviation of voice *f*_*o*_. Of the 18 total features (3 features currently in the aRFF and aRFF-AP algorithms plus 15 new acoustic features), 13 features were calculated directly from the microphone signal. This included autocorrelation, mean and standard deviation of cepstral peak prominence, cross-correlation, low-to-high ratio of spectral energy, normalized cross-correlation, normalized peak-to-peak amplitude, number of zero crossings, short-time energy, short-time log energy, short-time magnitude, signal-to-noise ratio, and waveform shape similarity. The remaining five features were calculated using a processed version of the microphone signal. Specifically, the third step of the aRFF-AP algorithm leverages the Auditory-SWIPE′ algorithm to calculate the *f*_*o*_ contour and pitch strength contour of each /ifi/ production. Three features were calculated from the *f*_*o*_ contour (average, median, and standard deviation of voice *f*_*o*_), and two features were computed using the pitch strength contour (average and median pitch strength).

In addition to examining the 13 acoustic features extracted from the raw microphone signal, filtered versions of these features were also considered. The aRFF and aRFF-AP algorithms employ a version of the microphone signal when band-pass filtered ±3 ST around the average *f*_*o*_ of the speaker to identify peaks and troughs in signal amplitude. The aRFF-AP algorithm also used this filtered version of the signal to compute normalized peak-to-peak amplitude (whereas the number of zero crossings and waveform shape similarity were calculated using the raw microphone signal). By including features computed from the filtered microphone signal, the result of this literature review resulted in a total of 31 acoustic features for subsequent analysis. Table 3 provides an overview of the acoustic features, including the signal used to calculate each and the proposed hypotheses in acoustic feature values when used for voiced/unvoiced detection.

##### 2.3.3.2 Feature Set Reduction

The 31-feature set was first examined to remove features that did not sufficiently capture the transition between voiced and unvoiced segments. The sliding window process in step 6 of the aRFF-AP algorithm was simulated to estimate each feature over time, ranging from the midpoint of the voiceless consonant and into the vowel. Acoustic feature trends were then examined relative to HSV-derived voicing transitions as a function of the number of pitch periods^2^ away from the “true” boundary cycle; specifically, the true boundary cycle was set to reference the time of voicing offset (t_off_) and the time of voicing onset (t_on_) to investigate the relationship between these acoustic features and the physiologically derived termination and initiation of vocal fold vibration, respectively. To comprehensively examine trends in feature values, the acoustic features were analyzed as a function of ±10 pitch periods from the true boundary cycle, resulting in 21 feature values (i.e., one feature value for each pitch period) for each of the 31 acoustic features per /ifi/. The feature values were then visually inspected to determine which acoustic features failed to exhibit a substantial change in feature magnitude during the transition between the voiceless consonant and vowel; such features were removed from subsequent analysis.

The remaining acoustic features were then used as predictors in a stepwise binary logistic regression to determine the probability of feature values corresponding to a voiced (1) or unvoiced (0) segment. The 21 values per acoustic feature for each of the 7721 /ifi/ productions were continuous predictors. Feature values were assumed independent in the regression model to identify which features were significantly related to voicing status rather than to create a regression equation for predicting voicing status. Variable significance was set to *p* < .05. Highly correlated features (variable inflation factor > 10) were removed from the model to reduce multicollinearity. Acoustic features that exhibited significant predictive effects and were sufficiently independent were retained for further algorithmic refinement.

##### 2.3.3.3 Algorithmic Modifications

The acoustic features that exhibited significant predictive effects on voicing status were introduced into the aRFF-AP algorithm to produce a more physiologically relevant version of the RFF algorithms called “aRFF-AP**H**” (aRFF-AP with **H**SV-derived acoustic features). The pitch strength rejection criterion of the aRFF-AP algorithm—which removes VCV productions with average pitch strength values below .05 from subsequent analysis due to little-to-no presence of a pitch sensation—was retained in the current study to streamline data processing. A sliding window based on the speaker’s estimated *f*_*o*_ then navigated from the voiceless consonant and into the vowel of interest. Within each window of time, the selected acoustic features from the current study were calculated rather than those within the aRFF-AP algorithm (i.e., normalized peak-to-peak amplitude, number of zero crossings, waveform shape similarity). Rule-based signal processing techniques were then adapted from the aRFF [21, 22] and aRFF-AP algorithms to identify the boundary cycle separating voiced and unvoiced segments. To locate this cycle, the algorithm identified a feature value that maximized the effect size between left and right components of each acoustic feature vector; the cycle index that corresponded to this identified feature value was selected as the boundary cycle candidate for that feature. From here, the median of these candidates was then calculated as the final boundary cycle.

#### 2.3.4 Performance of Manual and Semi-automated RFF Estimation Methods

To examine the impact of introducing physiologically relevant acoustic features into the semi-automated RFF algorithms, the ability of manual and algorithmic RFF estimation methods to locate the true boundary cycle (derived via HSV; referenced to t_off_ for voicing offset and t_on_ for voicing onset) was assessed. First, the 7721 /ifi/ productions from 122 participants were processed using the aRFF-AP and aRFF-APH algorithms as well as manual estimation techniques. The accuracy of the three RFF estimation methods was then quantified as the distance (in average pitch periods) between true and selected boundary cycles for each voicing offset and onset instance of each /ifi/ production. The distance between true and selected boundary cycles was compared across RFF estimation methods to determine which method best corresponded with vocal fold vibratory characteristics during intervocalic offsets and onsets.

### 2.4 Statistical Analysis

Chi-square tests were performed to determine whether there was a relationship between RFF estimation method (manual, aRFF-AP, aRFF-APH) and boundary cycle classification accuracy. Two chi-square tests were conducted: one for voicing offset and one for voicing onset. In each analysis, a correctly classified boundary cycle referred to an instance in which the distance between true and selected boundary cycles was zero (whereas a misclassified boundary cycle corresponded to some non-zero distance between true and selected boundary cycles). Significance was set *a priori* to *p* < .05. Cramer’s *V* was used to assess effect sizes of significant associations. Resulting effect sizes were interpreted using criteria from Cohen [49]. *Post hoc* chi-square tests of independence were then performed for pairwise comparisons of the three RFF estimation methods using a Bonferroni-adjusted *p* value of .017 (.05 / 3 comparisons).

## 3. Results

### 3.1 Acoustic Feature Trend Analysis

**Figure 3.**
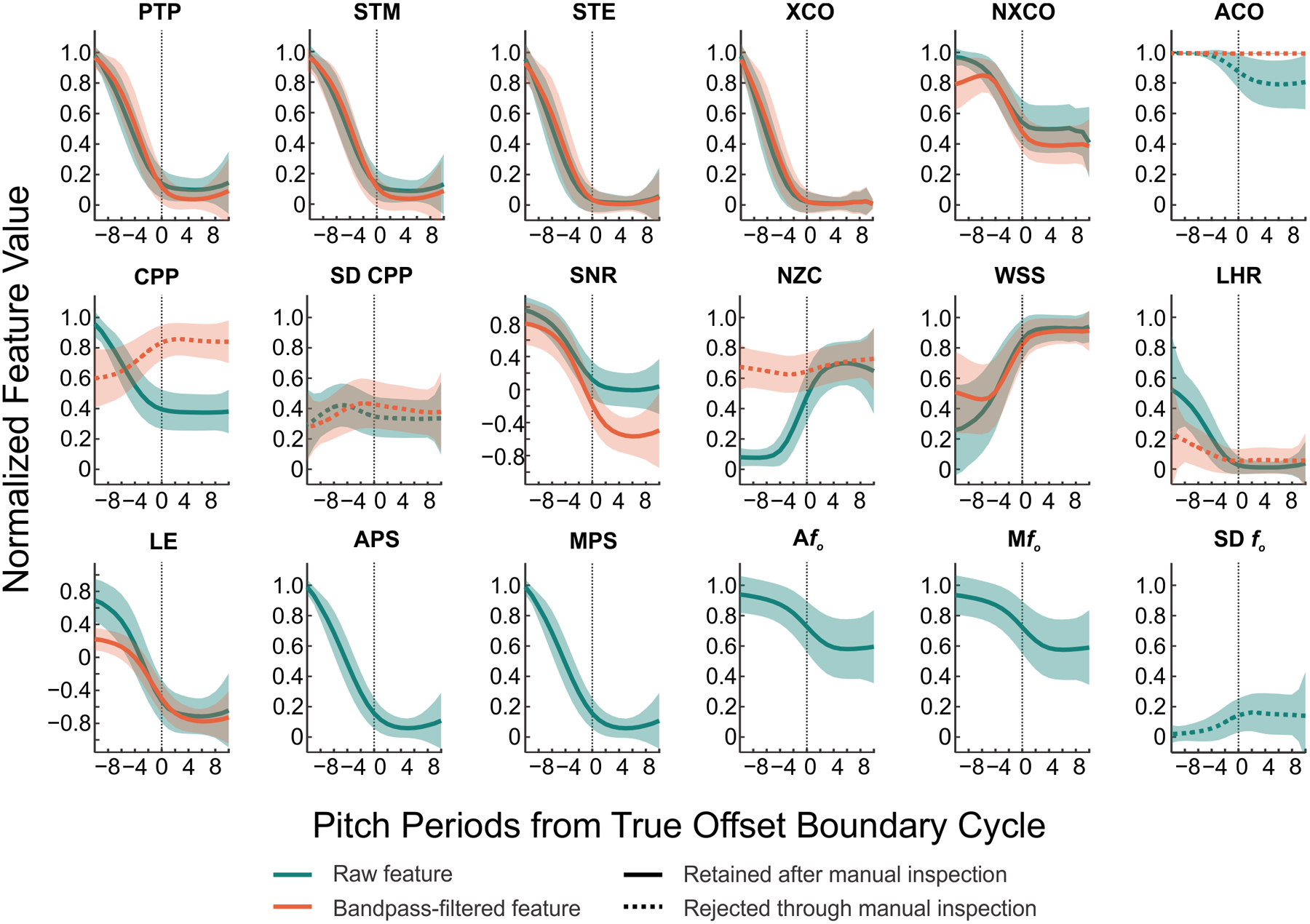
Normalized feature values calculated from the raw microphone signal or Auditory-SWIPE′ output (teal) with respect to distance (pitch periods) from the true boundary cycle (thin black dotted line) for voicing offset. Normalized feature values calculated from band-pass filtered microphone signal are overlaid in orange (when applicable). Top row: normalized peak-to-peak amplitude (PTP), short-time magnitude (STM), short-time energy (STE), cross-correlation (XCO), normalized cross-correlation (NXCO), autocorrelation (ACO). Middle row: mean and standard deviation of cepstral peak prominence (CPP, SD CPP), signal-to-noise ratio (SNR), number of zero crossings (NZC), waveform shape similarity (WSS), low-to-high ratio of spectral energy (LHR). Bottom row: log energy (LE), average and median pitch strength (APS, MPS), average, median, and standard deviation of *f*_*o*_ (A*f*_*o*_, M*f*_*o*_, SD *f*_*o*_). Thick solid lines indicate mean values of features that were retained after manual inspection. Thick orange and teal dashed lines indicate mean values of features that were removed through manual inspection. Shaded regions indicate standard deviation.

**Figure 4.**
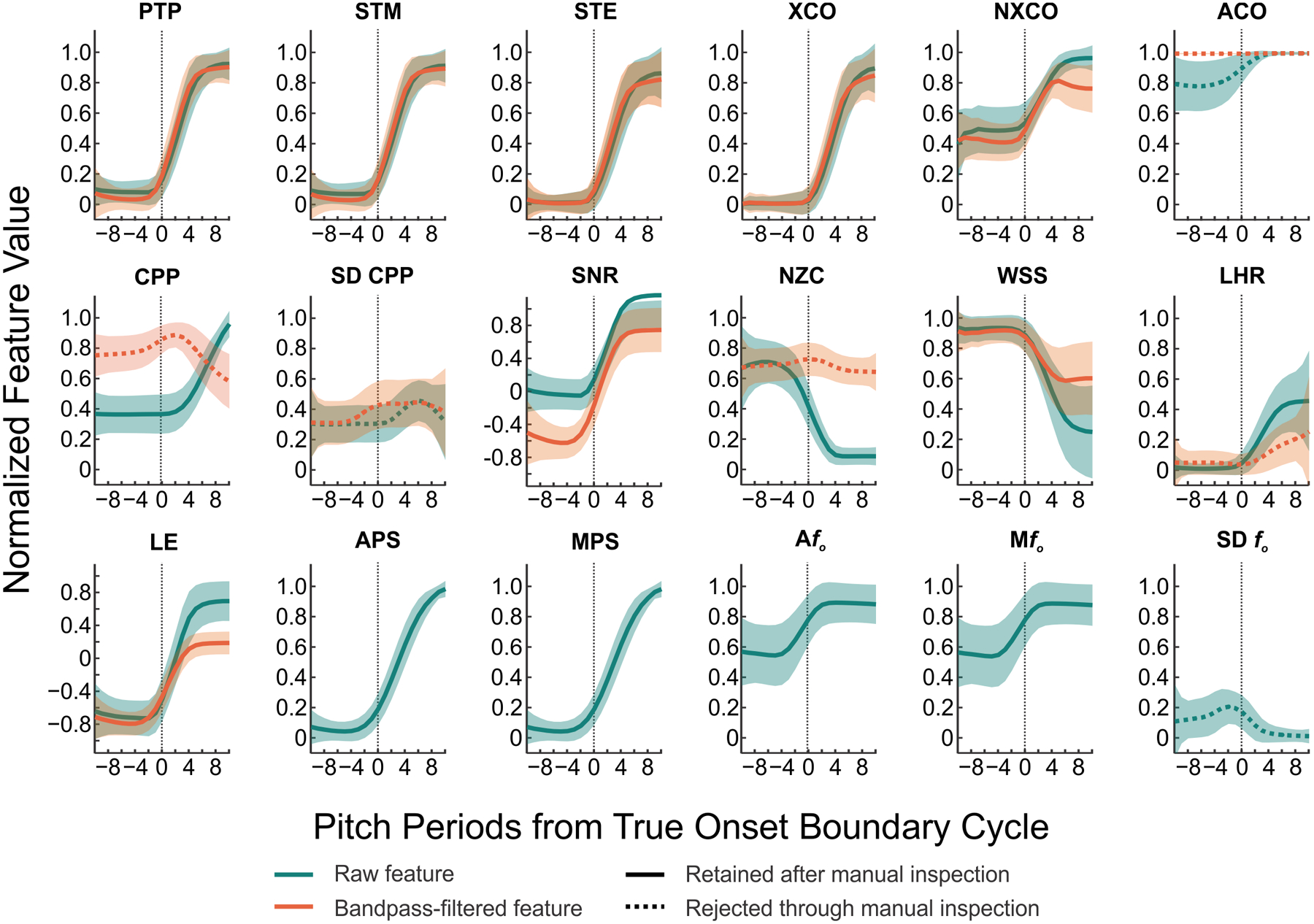
Normalized feature values calculated from the raw microphone signal or Auditory-SWIPE′ output (teal) with respect to distance (pitch periods) from the true boundary cycle (thin black dotted line) for voicing onset. Normalized feature values calculated from band-pass filtered microphone signal are overlaid in orange (when applicable). Top row: normalized peak-to-peak amplitude (PTP), short-time magnitude (STM), short-time energy (STE), cross-correlation (XCO), normalized cross-correlation (NXCO), autocorrelation (ACO). Middle row: mean and standard deviation of cepstral peak prominence (CPP, SD CPP), signal-to-noise ratio (SNR), number of zero crossings (NZC), waveform shape similarity (WSS), low-to-high ratio of spectral energy (LHR). Bottom row: log energy (LE), average and median pitch strength (APS, MPS), average, median, and standard deviation of *f*_*o*_ (A*f*_*o*_, M*f*_*o*_, SD *f*_*o*_). Thick solid lines indicate mean values of features that were retained after manual inspection. Thick orange and teal dashed lines indicate mean values of features that were removed through manual inspection. Shaded regions indicate standard deviation.

Fig. 3 shows the relationship between acoustic features and the true boundary cycle (relative to t_off_) for 7721 voicing offset instances. Fig. 4 shows this relationship (relative to t_on_) for 7721 voicing onset instances. Manual inspection of these 31 features resulted in the removal of the filtered number of zero crossings, raw and filtered autocorrelation, filtered cepstral peak prominence, filtered low-to-high ratio of spectral energy, raw and filtered standard deviation of cepstral peak prominence, and standard deviation of voice *f*_*o*_ due to a lack of discrimination between voiced and unvoiced segments (indicated by the dashed lines in Figs. 3 and 4). All further analyses were completed using the remaining 23 features (indicated by the solid lines in Figs. 3 and 4).

### 3.2 Acoustic Feature Set Reduction

The results of the logistic regression (shown in Table 4) indicated that filtered waveform shape similarity, median of voice *f*_*o*_, cepstral peak prominence, number of zero crossings, short-time energy, average pitch strength, normalized cross-correlation, and cross-correlation were all significant predictors of voicing status for voicing offset (*p* < .05). When using these eight features, the model for voicing offset accounted for 61.7% of the variance in voicing status (adjusted *R*^*2*^ = 61.7%), with an area under the receiver operating characteristic (ROC) curve of .96. Inspection of the coefficients indicated that the log odds of voicing decreased per one unit increase in short-time energy, normalized cross-correlation, number of zero crossings, or filtered waveform shape similarity (i.e., negative coefficient). On the other hand, the log odds of voicing increased per one unit increase in median of voice *f*_*o*_, cepstral peak prominence, average pitch strength, or cross-correlation (i.e., positive coefficient).

For voicing onset, the stepwise binary logistic regression revealed that filtered waveform shape similarity, median of voice *f*_*o*_, cepstral peak prominence, number of zero crossings, average pitch strength, signal-to-noise ratio, filtered short-time energy, and filtered short-time log energy were all significant predictors of voicing status (*p* < .05; see Table 4). The model for voicing onset accounted for 75.8% of the variance in voicing status (adjusted *R*^*2*^ = 75.8%), with an area under the ROC curve of .98. The model for voicing onset indicated that the log odds of voicing decreased per one unit increase in number of zero crossings or filtered short-time energy. The log odds of voicing increased per unit increase in filtered waveform shape similarity, median of voice *f*_*o*_, cepstral peak prominence, average pitch strength, signal-to-noise ratio, or filtered short-time log energy. The resulting acoustic features were then incorporated into the aRFF-APH algorithms to identify the boundary cycle of voicing.

### 3.3 Performance of Manual and Semi-automated RFF Estimation Methods

**Figure 5.**
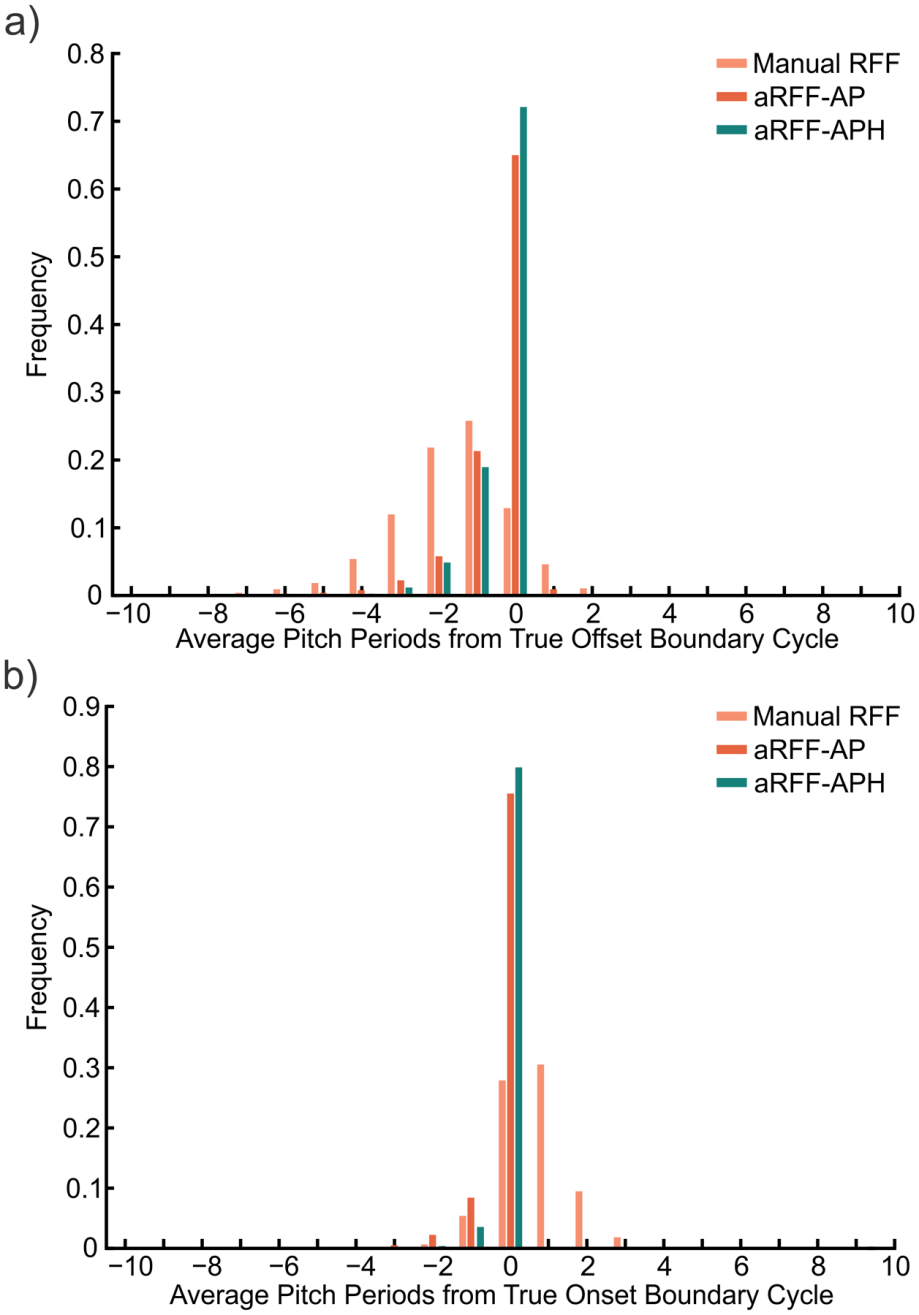
Boundary cycle identification of each RFF estimation method (manual, aRFF-AP, aRFF-APH). For (**a**) voicing offset and (**b**) voicing onset. Results for manual RFF estimation are shown in light orange, aRFF-AP in dark orange, and aRFF-APH in teal.

The comparison of aRFF-APH, aRFF-AP, and manual RFF estimation techniques in identifying the true boundary cycle is shown in Fig. 5. Out of 7721 offset instances (see Fig. 5a), the aRFF-APH algorithms correctly identified the boundary cycle in 72.1% of instances (*N* = 5565). The aRFF-AP algorithm correctly identified the boundary cycle in 65.0% of offset instances (*N* = 5021), followed by manual RFF estimation in which only 12.9% of offset boundary cycles (*N* = 994) were correctly identified. Misclassifications occurred at the rate of 25.6% for aRFF-APH (*N* = 1978), 32.2% for aRFF-AP (*N* = 2488), and 74.2% for manual RFF estimation (*N* = 5730). Nearly 13% of offset instances (*N* = 997) were rejected during manual estimation, whereas under 3% of offset instances were rejected by the aRFF-APH (*N* = 178) and aRFF-AP (*N* = 212) algorithms. For the algorithmic methods, three offset instances were automatically rejected due to pitch strength values < .05. The remainder of these rejections were due to errors in identifying voiced cycles (*N* = 151 for aRFF-AP, *N* = 150 for aRFF-APH), or post-processing of resulting RFF values (e.g., glottalization; *N* = 58 for aRFF-AP, *N* = 25 for aRFF-APH).

Out of 7721 onset instances (see Fig. 5b), the aRFF-APH algorithm correctly identified the boundary cycle in 80.0% of instances (*N* = 6170). The aRFF-AP algorithm correctly identified the boundary cycle in 77.2% of onset instances (*N* = 5833), followed by manual estimation in which 28.0% of onset instances (*N* = 2158) resulted in correctly identified boundary cycles. Misclassifications occurred at the rate of 4.0% for aRFF-APH (*N* = 310), 11.5% for aRFF-AP (*N* = 888), and 48.6% for manual RFF estimation (*N* = 3752). Almost a quarter (*N* = 1811) of onset instances were rejected during manual analysis. The aRFF-AP algorithm led to the least number of rejected onset instances (*N* = 1000; 13.0%), followed by the aRFF-APH algorithm (N = 1241; 16.1%). A total of 216 onset instances were automatically rejected by the aRFF-AP and aRFF-APH algorithms due to a pitch strength < .05; the remainder of these rejections were due to errors in identifying voiced cycles (*N* = 567 for aRFF-AP, *N* = 891 for aRFF-APH), or post-processing of resulting RFF values (*N* = 217 for aRFF-AP, *N* = 134 for aRFF-APH).

The results of the chi-square tests are shown in Table 5. Boundary cycle classification accuracy was significantly different for RFF estimation method, producing large effect sizes for both voicing offset (*p* < .001, *V* = .52) and onset (*p* < .001, *V* = .58). Boundary cycle classification accuracy was significantly different between manual and aRFF-AP methods. *Post hoc* analyses revealed a large effect for voicing offset (*p* < .001, *V* = .53) and onset (*p* < .001, *V* = .52), wherein aRFF-AP was more likely to correctly identify the boundary cycle than manual estimation. Boundary cycle classification accuracy was also significantly different between manual and aRFF-APH methods. *Post hoc* analyses showed a large effect for both voicing offset (*p* < .001, *V* = .59) and onset (*p* < .001, *V* = .62), such that aRFF-AP was more likely to correctly identify the boundary cycle. Finally, the boundary cycle classification accuracy was significantly different between semi-automated RFF algorithms (aRFF-AP, aRFF-APH) for both voicing offset and onset (*p* < .001); yet, the size of this effect was negligible for offset (*V* = .08) and small for onset (*V* = .15).

## 4. Discussion

The aim of the current study was to investigate the relationship between acoustic features and vocal fold vibratory characteristics during intervocalic voicing offsets and onsets. A large set of speakers with typical voices and speakers with voices characterized by excessive laryngeal muscle tension were instructed to produce the VCV utterance, /ifi/, while altering vocal rate and vocal effort. Simultaneous recordings were acquired using a microphone and flexible nasendoscope. The initiation (voicing onset) and termination (voicing offset) of vocal fold vibration were identified via inspection of the laryngoscopic images. A set of acoustic features were examined in reference to these time points, and a stepwise binary logistic regression was performed to identify which features best coincided with voicing offset and onset. The features that exhibited significant predictive effects were then implemented into the semi-automated RFF algorithm (“aRFF-APH”). The accuracy of the aRFF-APH algorithm in locating the transition between voiced and unvoiced segments was then assessed against (1) the current version of the semi-automated RFF algorithm (“aRFF-AP”), and (2) manual RFF estimation, the current gold-standard technique for calculating RFF.

The results of this investigation indicate that using the aRFF-APH algorithm led to the greatest percentage of correctly identified boundary cycles (76.0%), followed by the aRFF-AP algorithm (70.3%) then manual estimation (20.4%). This suggests that using physiologically tuned acoustic features to identify the transition between voiced and unvoiced segments—even in the absence of methods to account for differences in voice sample characteristics (i.e., as in the aRFF-AP algorithm)—improves the correspondence between algorithmic and physiologic boundary cycles. These findings are in support of our hypothesis that incorporating features related to the onset and offset of vocal fold vibration improves the accuracy of acoustic voiced/unvoiced boundary detection.

Although the aRFF-APH algorithm demonstrated greater accuracy in detecting voiced/unvoiced boundaries, the aRFF-AP algorithm remains the gold-standard method for semi-automatically estimating RFF. This is because the aRFF-AP algorithm was developed and validated using independent training and test sets to improve the clinical applicability of RFF. The aRFF-APH algorithm, on the other hand, was developed with the goal of improving the acoustic voiced/unvoiced detection rather than clinical applicability and was specifically tuned to the limited database examined here. As part of this investigation, all speakers were recorded in a sound-attenuated booth in the presence of constant noise from the endoscopic light source. In addition to this single recording location, the voice sample characteristics that were captured in the speaker dataset were more limited that those used in the development of the aRFF-AP algorithm: Vojtech, et al. [24] included over 20 different primary voice complaints with an overall severity of dysphonia ranging from 0 to 100, whereas the current study included a smaller range of diagnoses (57% typical, 16% MTD, 3% nodules, 2% polyp, 1% scarring, 1% lesion, 20% Parkinson’s disease) and resulting dysphonia severity (0–51.3). Because of the limited spectrum of vocal function captured here, pitch strength-tuned parameters and independent training/test sets were not implemented in the development of the aRFF-APH algorithm in the current study.

Despite the aforementioned differences between the aRFF-AP and aRFF-APH algorithms, using either of these methods resulted in a greater boundary cycle identification accuracy than when using manual estimation. These findings are unexpected since manual estimation has long been considered the gold-standard RFF estimation method, wherein trained technicians may exercise trial and error to identify the boundary cycle in difficult scenarios (e.g., poor recording environment and/or equipment, severe dysphonia) when cycle masking is present. It is possible that the characteristics of the speaker database confounded this outcome, as noise from the endoscopic light source may have masked the voice signals and/or speaker productions may have deviated from the norm due to the flexible nasendoscope. Even though manual estimation makes use of trial and error to subjectively locate the boundary cycle when masking is present, it is possible that manual estimation techniques were not sensitive enough to isolate the physiological boundary cycle in these conditions. On the other hand, the aRFF-AP algorithm was designed to account for such variations and the aRFF-APH algorithm was refined based on the physiologically determined vocal fold characteristics. Both algorithms also identify potential vocal cycles using a filtered version of the microphone signal that was designed to reduce the amplitude of vocal tract resonances, coarticulation due to concurrent frication and aspiration, and radiation of the lips. By only using the raw microphone signal to identify vocal cycles, the RFF values resulting from manual estimation may not reflect the true offset or onset of voicing as expected.

Although manual estimation resulted in the lowest boundary cycle identification accuracy, it is important to note that most misclassifications occurred within two pitch periods of the true boundary cycle for both voicing offset and onset (see Fig. 5). These findings are similar to those of Lien, et al. [50], in which manual RFF estimation was compared when performed on a microphone signal versus a neck-surface accelerometer signal. Since a neck-surface accelerometer is able to capture the vibrations of the glottal source in the absence of vocal cycle masking due to frication and aspiration [as may occur during the production of an intervocalic fricative; 27], the accelerometer signal was considered to be a ground-truth over the microphone signal. The authors observed that misclassifications occurred closer to the vowel for both voicing offset and onset when performing manual RFF estimation using a microphone signal rather than an accelerometer signal. Whereas offset RFF values were extracted approximately two cycles closer to the vowel when using a microphone signal, onset RFF values were computed less than one cycle away from the voiceless consonant when using a microphone signal. The results of the current study support these findings and, moreover, lend support to the supposition that the aRFF-AP and aRFF-APH algorithms benefit from using a band-pass filtered version of the microphone signal to identify potential vocal cycles.

As semi-automated RFF algorithm accuracy is typically quantified in reference to manual RFF estimation [e.g., see 22, 24], it is important to consider that manual estimation may not be a true gold-standard technique. Further investigation is necessary to examine the hypothesis that differences in boundary cycle identification may be attributed to the algorithms leveraging a band-pass filtered version of the microphone signal to reduce the impacts of vocal tract resonances, coarticulation due to concurrent frication and aspiration, and radiation of the lips. Such an investigation should include an analysis of both laryngeal imaging and acoustics to comprehensively assess the relevance and validity of manual estimation as the gold-standard technique for calculating RFF. Laryngeal imaging is a crucial component for this investigation, as this modality can provide physiological confirmation of vocal fold vibrations that are indirectly captured via RFF. In addition to comparing manual and semi-automated boundary cycle selections, this investigation should aim to compare the boundary cycles obtained via manual RFF estimation when using each version of the acoustic signal (i.e., microphone, accelerometer). In the event that manual estimation is no longer considered as gold-standard RFF method, efforts should be made to develop new metrics of algorithmic performance to replace those that are calculated in reference to RFF values obtained via manual estimation (e.g., root-mean-square error, mean bias error).

Even though manual RFF estimation demonstrated the lowest voiced/unvoiced boundary detection accuracy, it should also be noted that this method is currently the only means by which RFF can be calculated on running speech. Whereas manual RFF technicians are trained to process both isolated VCV productions and VCV productions extracted from running speech, the semi-automated RFF algorithm has been designed, trained, and tested on isolated VCV productions since its origination [see 22]. Thus, although aRFF-AP and aRFF-APH demonstrated greater accuracy in capturing the voicing transitions necessary to compute RFF, both versions of the algorithm can only be used in scenarios when the voice samples are compatible. It is therefore recommended that the aRFF-AP algorithm—which was validated across a broad spectrum of vocal function and recording locations [24]—be used in future investigations when compatible voice samples (i.e., isolated VCV productions) are available. In scenarios that require RFF to be computed from running speech, it is recommended that manual RFF estimation be used.

The results of the current study demonstrate the promise of using physiologically relevant acoustic features to locate the boundary cycle between voiced and unvoiced speech segments, specifically for estimates of RFF. However, additional steps must be undertaken to improve the clinical applicability of the aRFF-APH algorithm. This should include the use of independent training and test sets that span a broad range of vocal function. In doing so, the aRFF-APH algorithm could be modified to include pitch strength-tuned algorithm parameters to account for variations in voice sample characteristics. The aRFF-APH algorithms should also be expanded to neck-surface accelerometer signals, as there has been a growing interest in using the neck-surface vibrations generated during speech for ecological momentary assessment and ambulatory voice monitoring [e.g., 27, 51–60]. By capturing daily vocal behavior through a neck-surface accelerometer, vocal behaviors associated with excessive or imbalanced laryngeal muscle forces could be identified and monitored via RFF. Although an accelerometer-based RFF algorithm has been developed [61] future work should aim to improve this algorithm by identify physiologically tuned features that can be used to identify the true termination and initiation of vocal fold vibration. Doing so would further improve the clinical relevance of using RFF to assess and track laryngeal muscle tension.

## 5. Conclusions

The current study examined the relationship between acoustic outputs from the semi-automated RFF algorithm and physiological vocal fold vibratory characteristics during intervocalic offsets and onsets. By incorporating features that reflected the onset and offset of vocal fold vibration, algorithmic accuracy of voiced/unvoiced detection increased. Voiced/unvoiced boundary detection accuracy when using the RFF algorithm exceeded that of the gold-standard, manual method for calculating RFF. These findings highlight the benefits of incorporating features related to vibratory offsets and onsets for acoustic voiced/unvoiced boundary detection. It is recommended that the recently validated version of the semi-automated algorithm be used to calculate RFF when voice samples containing isolated vowel–voiceless consonant–vowel productions are available, and manual RFF estimation in scenarios that require RFF to be computed from running speech.

## Figures and Tables

**Table 1.** Overall demographic information for the 122 participants.

Cohort	Gender		Age			Overall Severity of Dysphonia		
	M	F	Mean	SD	Range	Mean	SD	Range
Young adults with typical voices	18	17	22.8	5.5	18–31	5.4	3.8	0.6–23.5
Older adults with typical voices	18	16	65.6	10.8	41–91	11.4	7.7	1.7–34.2
Adults with HVD^1^	6	22	37.5	16.1	19–70	12.3	10.7	0.9–38.5
Adults with PD^2^	18	7	63.0	9.4	43–75	19.2	13.3	4.0–51.3

1 HVD = Hyperfunctional voice disorder;

2 PD = Parkinson’s disease

**Table 2.** Number of participants for which each of five trained technicians manually computed relative fundamental frequency.^1^

Technician	Total Ratings	Number of Participants in Common between Technicians				
		1	2	3	4	5
1	37					
2	82	5				
3	79	18	53			
4	29	14	13	2		
5	17	0	11	6	0	

1 Total ratings sum to 244 as manual RFF estimation was performed twice for each participant (N=122).

**Table 3.** Acoustic measures for classifying voiced and unvoiced speech segments, with abbreviations (Abbr), the signal used to calculate the feature, feature definition, and proposed hypotheses surrounding feature trends shown. Rows that are shaded orange indicate that the acoustic feature was included in aRFF-AP algorithm.

Feature Name	Abbr.	Signal(s)	Definition	Hypothesized Feature Values in Voiced (V) vs. Unvoiced (UV) Segments
Autocorrelation	ACO	Raw and Filtered Microphone	ACO is a comparison of a segment of a voice signal to a delayed copy of itself as a function of the delay [40–42].	V > UV
Mean Cepstral Peak Prominence	CPP	Raw and Filtered Microphone	CCP reflects the distribution of energy at harmonically related frequencies [43] and is calculated as the magnitude of the peak with the highest amplitude in the cepstrum (i.e., the Fourier transform of the power spectrum).	V > UV
Average Pitch Strength	APS	Pitch Strength Contour	Using Auditory-SWIPE′ [25], pitch strength is calculated by correlating a voice signal with a sawtooth waveform constructed across a range of possible *f*_*o*_ values; the *f*_*o*_ value that elicits the greatest correlation is considered the *f*_*o*_ of the signal, and the degree of this correlation is the pitch strength. APS is then calculated as the average pitch strength of the window.	V > UV
Average Voice *f*_*o*_	A*f*_*o*_	*f*_*o*_ Contour	A*f*_*o*_ was calculated in the current study using the Auditory-SWIPE′ algorithm (described above in APS).	V > UV
Cross-correlation	XCO	Raw and Filtered Microphone	XCO is a comparison of a segment of a voice signal with a different segment of the signal [42, 44, 45].	V > UV
Low-to-high ratio of spectral energy	LHR	Raw and Filtered Microphone	LHR is calculated by comparing spectral energy above and below a specified frequency. Using a cut-off frequency of 4 kHz [43, 46], the LHR may distinguish harmonic energy of the /i/ from high-frequency aspiration and frication noise (above 2–3 kHz) of the /f/.	V > UV
Median Pitch Strength	MPS	Pitch Strength Contour	MPS was included as an alternative to APS.	V > UV
Median Voice *f*_*o*_	M*f*_*o*_	*f*_*o*_ Contour	M*f*_*o*_ was included as an alternative to A*f*_*o*_.	V > UV
Normalized Cross-correlation	NXCO	Raw and Filtered Microphone	NXCO was included as an alternative to XCO, in which the amplitude of the compared windows are normalized to remove differences in signal amplitude as a factor.	V > UV
Normalized Peak-to-peak Amplitude	PTP	Raw and Filtered Microphone	PTP is the range of the amplitude of a windowed voice signal.	V > UV
Number of Zero Crossings	NZC	Raw and Filtered Microphone	NZC refers to the number of sign changes of the windowed signal.	V < UV
Short-time Energy	STE	Raw and Filtered Microphone	STE is the energy of a short voice segment [41, 47, 48].	V > UV
Short-time Log Energy	SLE	Raw and Filtered Microphone	SLE was included as an alternative to STE, and is calculated as the logarithm of the energy of a short voice segment.	V > UV
Short-time Magnitude	STM	Raw and Filtered Microphone	STM is the magnitude of a short voice segment [41, 47, 48].	V > UV
Signal-to-noise Ratio	SNR	Raw and Filtered Microphone	SNR is an estimate of the power of a signal compared to that of a segment of noise.	V > UV
Standard Deviation of Cepstral Peak Prominence	SD CPP	Raw and Filtered Microphone	SD CPP is the standard deviation of CPP values within a window, and may capture variations in signal periodicity as a result of aspiration and frication noise in the /f/.	V < UV
Standard Deviation of Voice *f*_*o*_	SD *f*_*o*_	*f*_*o*_ Contour	SD *f*_*o*_ is the standard deviation of *f*_*o*_ values within a window, and may be subject to errors in *f*_*o*_ estimation during the /f/ (as unvoiced segments would not have a valid *f*_*o*_ value).	V < UV
Waveform Shape Similarity	WSS	Raw and Filtered Microphone	WSS is the normalized sum of square error between the current window of time and the previous window of time. It is calculated relative to a window of time in the voiceless consonant.	V < UV

**Table 4.** Summary of significant variables in the stepwise binary logistic regression statistical model.

Model	Acoustic Feature	Coef	SE Coef	*z*	*p*	95% Confidence Interval		VIF^1^
						Lower Bound	Upper Bound	
Voicing Offset	Constant	0.10	0.07	1.48	.15	−0.03	0.24	—
	Filtered Waveform Shape Similarity	−1.52	0.05	−30.07	<.001	−1.62	−1.42	1.30
	Median of Voice *f*_*o*_	1.46	0.04	34.85	<.001	1.37	1.54	1.21
	Cepstral Peak Prominence	1.23	0.06	20.07	<.001	1.11	1.35	1.27
	Number of Zero Crossings	−3.31	0.04	−78.69	<.001	−3.39	−3.23	1.55
	Short-time Energy	−5.72	0.15	−38.18	<.001	−6.01	−5.42	9.03
	Average Pitch Strength	9.24	0.12	78.52	<.001	9.01	9.47	4.81
	Normalized Cross-Correlation	−0.84	0.05	−16.77	<.001	−0.93	−0.74	1.53
	Cross-correlation	1.00	0.16	6.25	<.001	0.69	1.31	7.74
								
Voicing Onset	Constant	−2.18	0.10	−22.69	<.001	−2.37	−2.00	—
	Filtered Waveform Shape Similarity	1.40	0.08	18.34	<.001	1.25	1.55	1.30
	Median of Voice *f*_*o*_	2.21	0.06	40.31	<.001	2.10	2.31	1.19
	Cepstral Peak Prominence	1.05	0.08	12.53	<.001	0.89	1.22	1.06
	Number of Zero Crossings	−2.62	0.06	−42.15	<.001	−2.75	−2.50	1.66
	Average Pitch Strength	8.94	0.15	59.45	<.001	8.65	9.24	2.83
	Signal-to-noise Ratio	0.56	0.06	9.84	<.001	0.45	0.68	2.44
	Filtered Short-time Energy	−3.75	0.10	−37.51	<.001	−3.95	−3.56	3.66
	Filtered Short-time Log Energy	3.11	0.07	44.81	<.001	2.97	3.24	3.01

1 VIF = variable inflation factor

**Table 5.** Chi-square (*X*^***2***^) tests of independence to examine RFF estimation method and accuracy of boundary cycle identification for voicing offset and onset. Effect size interpretations of Cramer’s *V* are based on criteria from Cohen [49].

Model	RFF Estimation Methods	*df*	*N*	*X* ^*2*^	*p*	*V*	Effect Size Interpretation
Voicing Offset	Manual vs. aRFF-AP vs. aRFF-APH	2	21793	5821.0	<.001	.52	Large
	Manual vs. aRFF-AP	1	14250	3928.0	<.001	53	Large
	Manual vs. aRFF-APH	1	14268	4982.0	<.001	.59	Large
	aRFF-AP vs. aRFF-APH	1	15068	89.7	<.001	.08	Negligible
							
Voicing Onset	Manual vs. aRFF-AP vs. aRFF-APH	2	19112	6417.0	<.001	.58	Large
	Manual vs. aRFF-AP	1	12631	3420.0	<.001	.52	Large
	Manual vs. aRFF-APH	1	12391	4831.0	<.001	.62	Large
	aRFF-AP vs. aRFF-APH	1	13202	283.0	<.001	.15	Small

## Data Availability

The data presented in this study are available on request from the corresponding author. The data are not publicly available due to the identifiable nature of voice acoustic recordings.

## Footnotes

1 The dataset used to train individuals in manual relative fundamental frequency estimation is a separate dataset from that described here and may be downloaded from https://sites.bu.edu/stepplab/research/rff/ (Last viewed August 6, 2020). Technicians were required to meet an interrater reliability criterion ≥ .93, as described in Vojtech, et al. [24].

2 “Pitch period” refers to the duration of one glottal cycle, and was computed per /ifi/ production using the average *f*_*o*_ determined using Auditory-SWIPE′.
